# Appropriateness of antibiotic prescribing varies by clinical services at United States children’s hospitals

**DOI:** 10.1017/ice.2023.56

**Published:** 2023-11

**Authors:** Devin T. Diggs, Alison C. Tribble, Rebecca G. Same, Jason G. Newland, Brian R. Lee

**Affiliations:** 1 College of Science, University of Notre Dame, Notre Dame, Indiana; 2 Division of Pediatric Infectious Diseases, Department of Pediatrics, University of Michigan, Ann Arbor, Michigan; 3 Division of Infectious Diseases, Department of Pediatrics, Children’s Hospital of Philadelphia; 4 Perelman School of Medicine at the University of Pennsylvania, Philadelphia, Pennsylvania; 5 Division of Pediatric Infectious Diseases, Department of Pediatrics, Washington University in St. Louis, St. Louis, Missouri; 6 Division of Health Services and Outcomes Research, Department of Pediatrics, Children’s Mercy Kansas City, Kansas City, Missouri

## Abstract

**Objective:**

To describe patterns of inappropriate antibiotic prescribing at US children’s hospitals and how these patterns vary by clinical service.

**Design:**

Serial, cross-sectional study using quarterly surveys.

**Setting:**

Surveys were completed in quarter 1 2019–quarter 3 2020 across 28 children’s hospitals in the United States.

**Participants:**

Patients at children’s hospitals with ≥1 antibiotic order at 8:00 a.m. on institution-selected quarterly survey days.

**Methods:**

Antimicrobial stewardship physicians and pharmacists collected data on antibiotic orders and evaluated appropriateness of prescribing. The primary outcome was percentage of inappropriate antibiotics, stratified by clinical service and antibiotic class. Secondary outcomes included reasons for inappropriate use and association of infectious diseases (ID) consultation with appropriateness.

**Results:**

Of 13,344 orders, 1,847 (13.8%) were inappropriate; 17.5% of patients receiving antibiotics had ≥1 inappropriate order. Pediatric intensive care units (PICU) and hospitalists contributed the most inappropriate orders (n = 384 and n = 314, respectively). Surgical subspecialists had the highest percentage of inappropriate orders (22.5%), and 56.8% of these were for prolonged or unnecessary surgical prophylaxis. ID consultation in the previous 7 days was associated with fewer inappropriate orders (15% vs 10%; *P* < .001); this association was most pronounced for hospitalist, PICU, and surgical and medical subspecialty services.

**Conclusions:**

Inappropriate antibiotic use for hospitalized children persists and varies by clinical service. Across 28 children’s hospitals, PICUs and hospitalists contributed the most inappropriate antibiotic orders, and surgical subspecialists’ orders were most often judged inappropriate. Understanding service-specific prescribing patterns will enable antimicrobial stewardship programs to better design interventions to optimize antibiotic use.

A majority of hospitalized children receive antibiotics during their admission.^1,2^ Inappropriate antibiotic prescribing contributes to the emergence of resistant pathogens and adverse drug events and may also result in treatment failure.^3–6^ A 2016–2017 point-prevalence survey of antibiotic use in hospitalized US children, conducted by 32 Sharing Antimicrobial Reports for Pediatric Stewardship (SHARPS) Collaborative hospitals, documented that 35% of admitted children had at least 1 active antibiotic order at any point in time.^7,8^ Of these patients, 26% received antibiotics that were considered suboptimal by antimicrobial stewardship programs (ASPs). This study also reported that routine ASP practices would not have reviewed nearly half of these suboptimal antibiotic orders. Additional characterization of inappropriate antibiotic prescribing is needed to target pediatric antimicrobial stewardship interventions to further improve antibiotic prescribing. To address this gap, the SHARPS Collaborative revised its point-prevalence survey to capture additional facets of antibiotic prescribing. This study further describes patterns of inappropriate prescribing, with a focus on the impact of clinical services.

## Methods

### Study design, setting, and population

Data for this study were derived from a point-prevalence survey completed by the SHARPS Collaborative. The methodology and findings from the original survey were previously published.^7^ In this iteration, 28 hospitals participated, and each hospital completed up to 7 quarterly surveys from January 2019 to September 2020. Institutional review board (IRB) approval was obtained from Children’s Mercy–Kansas City (CMH, the coordinating center) and at hospitals that did not adopt central IRB approval through CMH. The study population consisted of patients admitted to the children’s hospital or pediatric ward at 8:00 a.m. on the day of each quarterly survey with an active order for an enteral, parenteral (intravenous, intramuscular, intrathecal, intraperitoneal), or inhaled antimicrobial.

### Data collection

For all eligible antimicrobial orders, ASP physicians and/or pharmacists classified whether antimicrobial use was inappropriate (“yes” or “no”), using the following standardized definition for inappropriate use: “The patient should not be receiving this drug via this route at this time.” For inappropriate antimicrobials, additional detail on why the antimicrobial was considered inappropriate was collected using fourteen prespecified reasons (Supplementary Table 1 online); “other” included any additional reasons that antimicrobial use was considered inappropriate, based on ASP site-specific practices, and ASP members elaborated the reasoning using a free-text explanation. Only 1 inappropriate reason could be chosen for each antimicrobial, and ASP physicians and pharmacists were instructed to select the most important reason.

Additionally, the survey collected the patient’s clinical service and whether the patient’s chart had a consultation note from an infectious diseases (ID) physician within the previous 7 days.

### Data analysis

Due to the emergence of COVID-19 in 2020, data were stratified by calendar year to assess differences in patient demographic data, frequency of drug use, and distribution of clinical services potentially attributable to pandemic-related changes in pediatric hospitalizations. No meaningful differences were identified (Supplementary Tables 2 and 3 online), so 2019 and 2020 data were pooled for analysis. After initial analysis of antimicrobial prevalence, all further analyses were restricted to antibiotics prescribed for infectious use (treatment or prophylaxis), excluding drugs prescribed for noninfectious indications. The primary outcome was the percentage of inappropriate antibiotic use, stratified by antibiotic class and clinical service. Antibiotics and clinical services were grouped for analysis (Supplementary Tables 4 and 5 online). Secondary outcomes included frequencies of reasons for inappropriate use and association of ID consultation with appropriate antibiotic prescribing. The Pearson χ^2^ test was used for categorical tabulations. All analyses were completed using R version 4.2.1 software (R Foundation for Statistical Computing, Vienna, Austria).

## Results

### Study population and antibiotic prevalence

In total, 28 hospitals contributed ≥1 quarter of survey data; 5 hospitals responded in all 7 quarters. Of 28,987 patients admitted across all survey days, 10,375 (35.8%) had ≥1 active antimicrobial order. In total, 18,389 active antimicrobial orders for both infectious and noninfectious indications occurred across all surveyed quarters. Antibiotics accounted for 78.2% (n = 14,386) of these orders. Infectious indications accounted for 13,672 antibiotic orders (95.0%), and further analysis was limited to these orders.

**Fig. 1.**
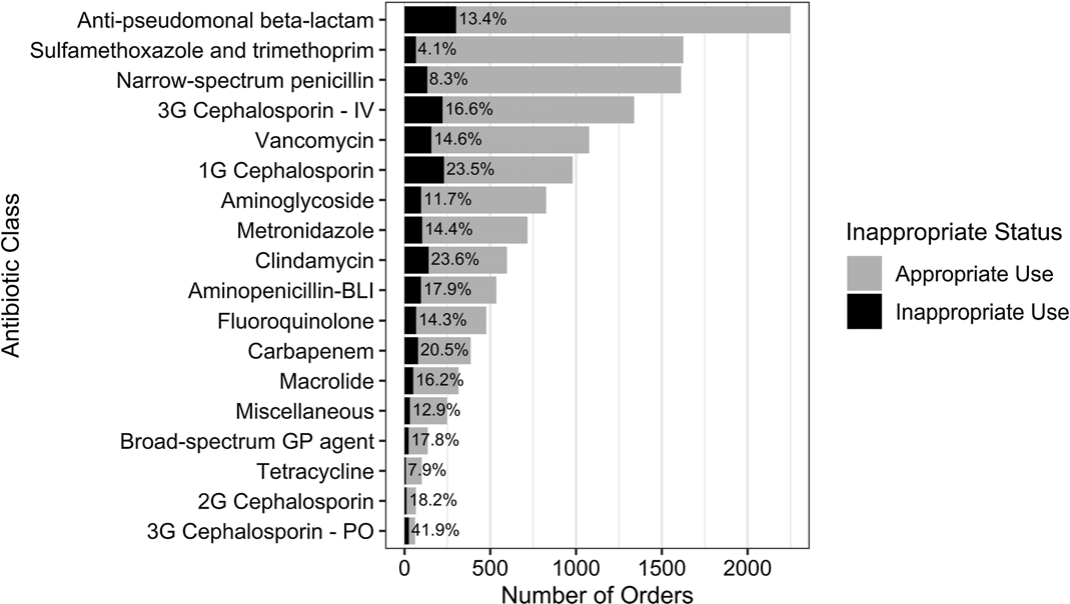
Antibiotic classes most frequently ordered for hospitalized children. The number within each bar indicates the percentage of inappropriate use within the antibiotic class. Note. 3G, third generation; IV, intravenous; 1G, first generation; BLI, β-lactam inhibitor; GP, gram positive; 2G, second generation; PO, oral.

Among the 9,263 patients receiving antibiotics, 5,930 (64.0%) patients had 1 active antibiotic order; 2,730 patients (29.5%) had 2 active orders; and 603 patients (6.5%) had ≥3 active orders. The median age was 4.0 years (interquartile range, 0.0–12.0 years), and 4,937 (53.3%) were male (Table 1). By number of patients enrolled, the top quartile of hospitals contributed 49.9% of patients, and the bottom quartile contributed 7.9%. Patients were predominantly cared for by the following services: hematology-oncology (19.1%), hospitalists (18.8%), pediatric intensive care unit (PICU, 14.7%), neonatal intensive care unit (NICU, 12.7%), and general surgery (6.9%). Antipseudomonal β-lactams (n = 2,249) and trimethoprim-sulfamethoxazole (TMP-SMX, n = 1,625) were the most frequently prescribed antibiotic classes (Fig. 1).

**Table 1.** Demographic and Clinical Characteristics of Hospitalized Children Receiving Antibiotics

Variable	Patients Receiving ≥ 1 Inappropriate Orders, (N=1,622), No. (%)^a^	Patients Receiving Appropriate Orders, (N=7,641), No. (%)^a^	All Patients, (N=9,263), No. (%)^a^
Patient age, median y (IQR)	4.0 (0.0–12.0)	4.0 (0.0–12.0)	4.0 (0.0–12.0)
**Sex**			
Female	777 (18)	3,507 (82)	4,284
Male	838 (17)	4,099 (83)	4,937
**Clinical service**			
CICU	117 (19)	510 (81)	627
Hospitalists	292 (17)	1,433 (83)	1,725
General surgery	128 (20)	508 (80)	636
Hematology-oncology	195 (11)	1,557 (89)	1,752
Medical subspecialties	249 (19)	1,091 (81)	1,340
NICU	144 (12)	1,022 (88)	1,166
PICU	330 (24)	1,017 (76)	1,347
Surgical subspecialties	152 (26)	442 (74)	594
**Ventilation status**			
Invasive ventilation	358 (22)	1,254 (78)	1,612
No ventilation	1,023 (16)	5,348 (84)	6,371
Noninvasive ventilation	230 (19)	992 (81)	1,222
Unknown	6 (27)	16 (73)	22
**Infectious disease consultation**			
No	1,360 (19)	5,934 (81)	7,294
Yes	252 (13)	1,639 (87)	1,891
**Immunocompromised status**			
No	1,338 (20)	5,419 (80)	6,757
Yes	281 (11)	2,202 (89)	2,483

Note. IQR, interquartile range; PICU, pediatric intensive care unit; CICU, cardiac intensive care unit.

a Units unless otherwise specified.

Assessment for inappropriate use was performed on 13,344 antibiotic orders (97.6%). Of these, 1,847 (13.8%) were considered inappropriate, and 17.5% of patients receiving antibiotics were prescribed ≥1 inappropriate antibiotic order. The percentage of antibiotics judged inappropriate did not vary significantly between hospitals that completed 5 or more quarters of the survey vs those that did not (14.0% vs 13.3%; *P* = .36), by season (14.2%, 13.9%, 12.9%, and 14.8% for Q1, Q2, Q3, and Q4, respectively; *P* = .17), or by year (16.5% vs 14.9% for 2019 and 2020, respectively; *P* = .07). The PICU and hospitalists cared for the most patients receiving at least 1 inappropriate antibiotic: 330 (21.0%) and 292 (18.0%) of 1,622 patients, respectively.

### Inappropriate use by clinical service

**Fig. 2.**
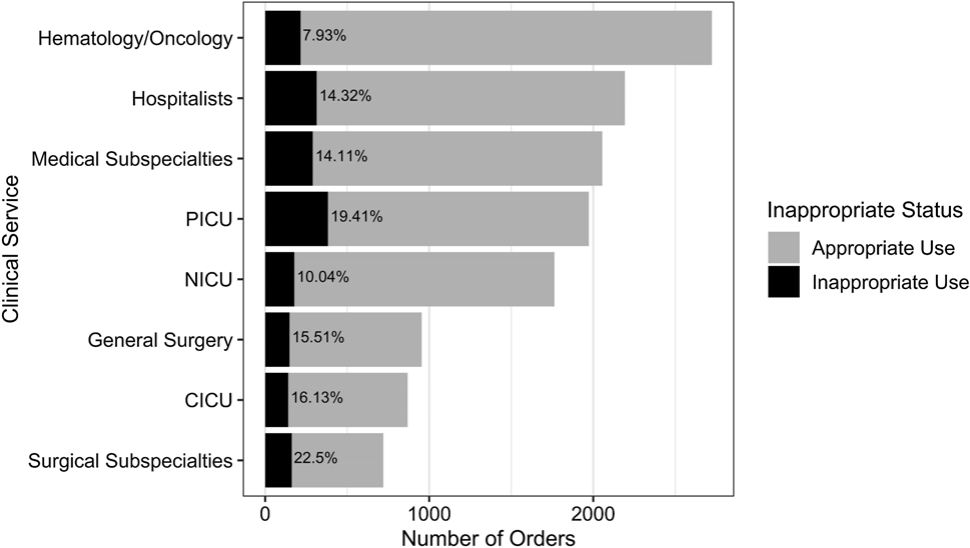
Frequency of antibiotic orders for hospitalized children. The number within each bar indicates the percentage of inappropriate use within the respective clinical service. Note. PICU, pediatric intensive care unit; CICU, cardiac intensive care unit.

**Fig. 3.**
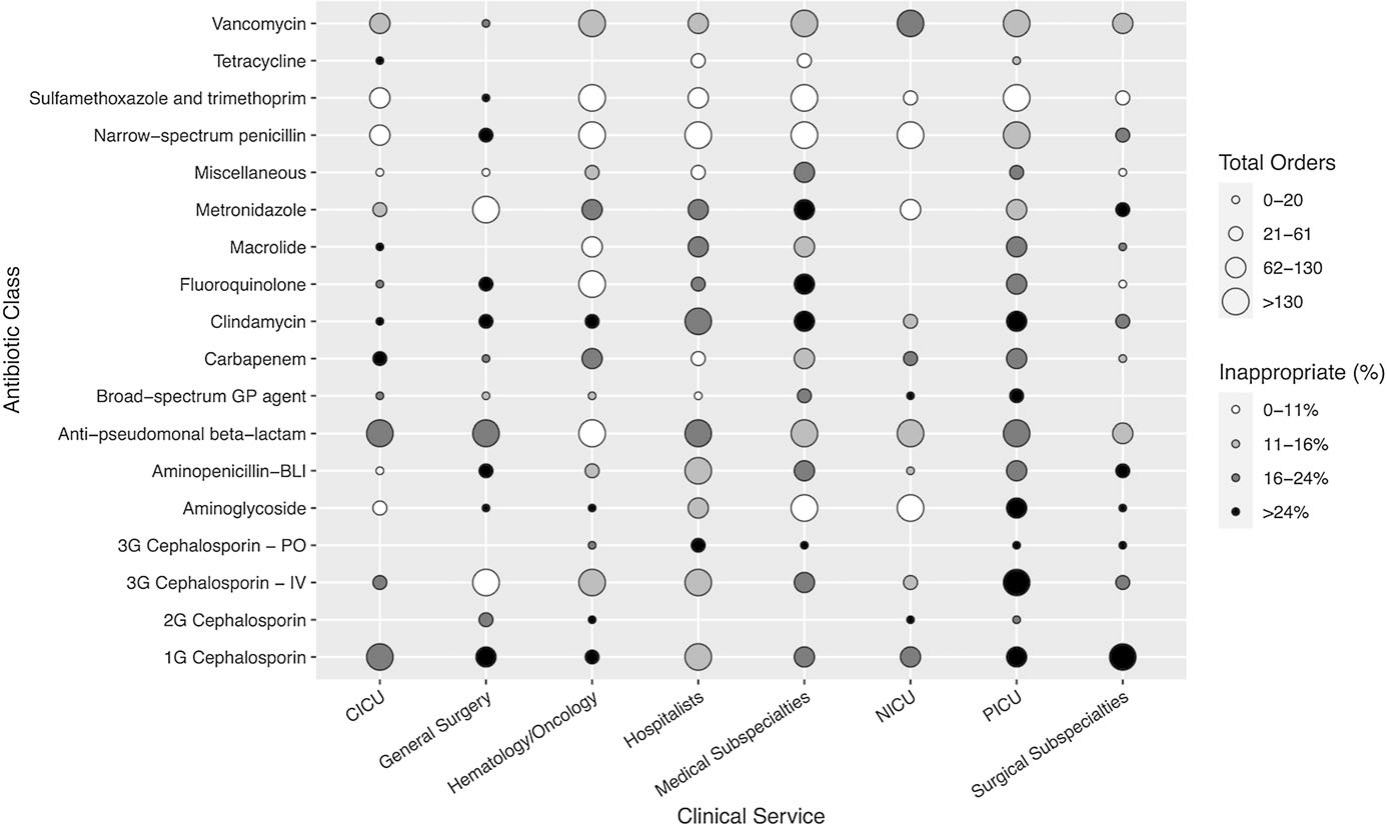
Heat map indicating antibiotic use by clinical service and antibiotic class. The total number of orders and percentage of inappropriate orders was calculated for each antibiotic classification and clinical service combination (Supplementary Table 6 online). The size of the point represents the number of antibiotic orders placed by each service. Each point size represents 1 quartile of the distribution of total orders by antibiotic classification and clinical service combination. The percentage of inappropriate orders determines the color of the points. Each of the 4 colors approximately represents 1 quartile of the distribution of percentages by antibiotic classification and clinical service combination, where white represents the first quartile and black represents the fourth quartile. Note. 1G, first generation; 2G, second generation; 3G, third generation; BLI, β-lactam inhibitor; CICU, cardiac intensive care unit; GP, gram positive; IV, intravenous; PICU, pediatric intensive care unit; PO, oral.

Across all clinical services, the PICU had the highest number of inappropriate antibiotic orders (n = 383 orders, 19.4%) (Fig. 2). Of these inappropriate orders, 21.6% were classified as inappropriate due to ASP assessment that treatment for bacterial infection was not indicated, and 30.0% were deemed unnecessarily broad spectrum (Table 2). Within the PICU, 24.7% of intravenous third-generation cephalosporins were classified as inappropriate and accounted for 73 (19.0%) of 383 inappropriate antibiotic orders. The antibiotics with the highest percentage of inappropriate prescriptions within the PICU were clindamycin (27 of 89 orders, 30.3%), followed by first-generation cephalosporins (33 of 120 orders, 27.5%) and aminoglycosides (21 of 81 orders, 25.9%) (Fig. 3).

**Table 2.** Leading Reasons for Inappropriate Antibiotic Use by Clinical Service

Service	Reason for Inappropriate Use	No. of Orders, n/N (%)^a^
PICU	Treatment not indicated	83/383 (21.7)
	Too broad based on culture or susceptibility	58/383 (15.1)
	Too broad empirically	57/383 (14.9)
Hospitalists	Too broad empirically	64/314 (20.4)
	Treatment not indicated	59/314 (18.8)
	Too broad based on culture or susceptibility	51/314 (16.2)
Surgical Subspecialties	Prolonged surgical prophylaxis	74/162 (45.7)
	Prophylaxis not indicated	18/162 (11.1)
	Too broad based on culture or susceptibility	11/162 (6.8)
Medical Subspecialties	Too broad based on culture or susceptibility	52/290(17.9)
	Too broad empirically	37/290 (12.8)
	Treatment not indicated	37/290 (12.8)

Note. PICU, pediatric intensive care unit.

a % indicates the percentage of inappropriate orders from the service thatwere due to the reason in the corresponding row.

Hospitalists contributed the second highest number with 314 inappropriate orders (17.0%), and 14.3% of all hospitalist orders were classified as inappropriate. Intravenous third-generation cephalosporins (61 of 409 orders, 14.9%) and clindamycin (53 of 271 orders, 19.6%) accounted for the largest number of inappropriate hospitalist orders, while, by proportion, inappropriateness was highest among oral third-generation cephalosporins (10 of 23 orders, 43.5%) and metronidazole (19 of 93 orders, 20.4%).

Medical subspecialties ordered 2,055 antibiotics, with 14.1% (290 orders) considered inappropriate. Pulmonary services contributed the most inappropriate orders (140 of 940 orders, 14.9%), mostly consisting of aminoglycosides (26 of 219 orders, 11.9%), antipseudomonal β-lactams (17 of 197 orders, 8.63%), and intravenous third-generation cephalosporins (11 of 40 orders, 27.5%). Gastroenterology contributed the second highest number of inappropriate orders (66 of 311 orders, 21.2%), with antipseudomonal β-lactams (19 of 77 orders, 24.7%) and vancomycin (13 of 57 orders, 22.8%) most often inappropriately prescribed.

Surgical subspecialties had the highest percentage of inappropriate use (22.5%). Across individual subspecialties, urology had the highest percentage of inappropriate antibiotic use at 33.3% (24 of 72 orders), followed by plastic surgery at 28.9% (22 of 76 orders), otolaryngology at 28.6% (16 of 56 orders), and neurosurgery at 24.1% (47 of 195 orders). Nearly half of inappropriate orders from surgical subspecialties were first-generation cephalosporins (80 of 162 orders, 49.4%).

Hematology-oncology (H-O) services had the lowest rate of inappropriate use (7.93%). Sulfamethoxazole and trimethoprim and antipseudomonal β-lactams were the most frequently ordered H-O antibiotics and were rarely classified as inappropriate: 8 (0.90%) of 884 orders and 42 (6.20%) of 678 orders, respectively. A full table of percentages of inappropriate orders by antibiotic classification and clinical service is provided in Supplementary Table 6 (online).

### Reasons for inappropriate use

The services contributing the greatest number of inappropriate antibiotic orders (PICU, hospitalists, and medical subspecialists) frequently had the same leading reasons for inappropriate use: treatment for bacterial infection was not indicated, or the spectrum was too broad either empirically or was based on results of cultures, antibiotic susceptibility testing, or rapid diagnostics (Table 2). Conversely, most inappropriate orders from surgical subspecialties were due to prolonged or unnecessary surgical prophylaxis (Table 2).

The greatest number of inappropriate prescriptions were for antipseudomonal β-lactams (301 of 2,249 orders, 13.4%). Across all services, most inappropriate antipseudomonal β-lactam orders were due to the agent being too broad-spectrum, either empirically or based on culture/susceptibility data (n = 159, 52.8%). Unnecessarily prolonged treatment duration was the next most common reason (n = 45, 15.0%).

First-generation cephalosporins were the second most common inappropriately prescribed antibiotics, contributing 12.5% of total inappropriate orders. Across all services, 133 (57.8%) of all 230 inappropriate first-generation cephalosporin orders were due to unnecessarily prolonged surgical prophylaxis, followed by no prophylaxis indicated (n = 35, 15.2%). Of 893 antibiotic orders for surgical prophylaxis, 32.2% were considered inappropriate.

In 91 inappropriate orders for intravenous third-generation cephalosporins (41%), antibiotics were unnecessary (treatment for infection was not indicated, indication for use was unclear, or unnecessarily prolonged treatment duration). An additional 92 inappropriate intravenous third-generation cephalosporin orders (41.4%) were considered inappropriate due to the spectrum being too broad.

Overall, 23.6% of clindamycin orders were considered inappropriate, with most inappropriate orders prescribed by PICUs and hospitalists (80 of 141 orders, 56.7%). Across all services, the leading reasons for inappropriate clindamycin use were that spectrum of the agent was too broad (n = 44, 31.2%) and that the drug could have been administered orally rather than intravenously (n = 26, 18.4%).

### Infectious diseases consultation

Recent ID consultation, signified by a note from an ID physician within the past 7 days, was documented for 3,047 (23.0%) of all 13,234 antibiotic orders. Overall, inappropriate use was significantly less common when ID had recently consulted (10.0% vs 15.0%; *P* < .001) (Table 3). However, the percentage of inappropriate orders did not differ with an ID consultation within the NICU, hematology-oncology, the cardiac intensive care unit, and general surgery services.

**Table 3.** Percentage of Inappropriate Orders by Presence of ID Consult and Service

Service	Percentage of Inappropriate Orders		
	No ID Consultation, n/N (%)	ID Consultation, n/N (%)	*P* Value
All services	1,530/10,187 (15.0)	304/3,047 (10.0)	<.001
NICU	146/1,448 (10.1)	31/304 (10.2)	1
Hematology-oncology	171/2,211 (7.7)	42/490 (8.6)	.60
CICU	106/630 (16.8)	34/227 (15.0)	.59
General surgery	137/845 (16.2)	11/101 (10.9)	.21
Hospitalists	258/1,515 (17.0)	56/662 (8.5)	<.001
PICU	296/1,270 (23.3)	85/681 (12.5)	<.001
Surgical subspecialties	148/546 (27.1)	9/163 (5.5)	<.001
Medical subspecialties	252/1,647 (15.3)	35/399 (8.8)	.001

Note. NICU, neonatal intensive care unit; CICU, cardiac intensive care unit; PICU, pediatric intensive care unit.

For surgical subspecialties, only 5 of 333 antibiotics prescribed for surgical prophylaxis were for patients with ID consultation.

## Discussion

Utilizing the national SHARPS Collaborative, this study further characterizes antibiotic use data from US children’s hospitals, adding important detail about prescribing variation by clinical service. Overall antibiotic prevalence remains stable, with 34.0% of patients actively receiving antibiotics in this survey compared to 35.0% in the 2016–2017 SHARPS survey and 36.3% in a 2017–2018 study of inpatient antibiotic use in US children’s hospitals.^7,9^

Of 13,344 total antibiotic orders for infectious indications, 13.8% were deemed inappropriate by ASP physicians and pharmacists. This result cannot be directly compared to the 2016–2017 study because inappropriate use was not explicitly defined in that study.^7^ However, for nearly all suboptimal orders in that study, ASP clinicians indicated how they would modify the order, and we estimate that 15.4%–18.3% of orders would have been classified as inappropriate with the updated definition. Thus, inappropriate use is likely stable to slightly reduced since the prior survey.

Visualizing antibiotic orders by clinical service and antibiotic class in the heat map revealed considerable variability in prescribing volume and appropriateness, identifying actionable targets and providing a new framework to guide refined ASP interventions. For example, the PICU and hospitalists contributed the highest number of inappropriate antibiotic orders, and these inappropriate orders were spread across multiple antibiotic classes. Third-generation cephalosporins, clindamycin, and aminoglycosides were prescribed inappropriately at rates of 25% or higher in the PICU. The volume and breadth of inappropriate orders among hospitalists and the PICU suggests these services may benefit from broad, service-wide interventions. The PICU and hospitalists also shared common reasons for inappropriate use: treatment for infection was not indicated or the spectrum was too broad. Studies have found that low risk tolerance and diagnostic uncertainty drive overuse of broad-spectrum antibiotics among intensivists and hospitalists, which may partly explain these findings.^10–12^ ASPs should consider these concerns and explore institutional service-level data to optimally design interventions to improve antibiotic use in these settings.

Across all services, 1 in 3 orders for surgical prophylaxis was classified as inappropriate, unchanged from the first SHARPS study.^13^ More than half of surgical subspecialists’ inappropriate orders were due to prolonged or unnecessary surgical prophylaxis, contributing to surgical subspecialties having the highest percentage of inappropriate orders at 22.5%. Unlike the PICU and hospitalists’ inappropriate use across antibiotic classes, inappropriate orders from surgical subspecialties were concentrated among first-generation cephalosporins. For these services, development and implementation of prophylaxis guidelines and/or targeted review of first-generation cephalosporins may be most effective in reducing inappropriate antibiotic use.

Prior interventional and observational studies have shown that ID consultation in the acute-care setting is associated with reduction of antibiotic use and improvement in antibiotic appropriateness.^14,15^ This study replicated the association between ID consultation and reduced inappropriate prescribing and additionally demonstrated that the association varies by clinical service. Specifically, only hospitalists, the PICU, and surgical and medical subspecialties had significantly fewer inappropriate orders following ID consultation. Some of these differences may be driven by service-level variation in patient types (eg, complicated infections) or protocolized care (eg, hematology-oncology). Additionally, surgical prophylaxis orders were rarely associated with ID consultation and comprised the majority of surgical subspecialists’ prescribing and inappropriate antibiotic use. However, differential uptake of ID recommendations by service is also possible, with adherence to ID recommendations ranging from 35% to 90% in prior studies.^14,16–19^ These findings may represent opportunities to improve antibiotic prescribing through increased ID consultation and/or enhanced uptake of ID recommendations. Notably, 10.0% of orders prescribed within 7 days of an ID consultation were still considered inappropriate, suggesting there may be opportunity for improvement among ID consultants as well.

Strengths of this study include multicenter and multiseasonal data collection, a clearly defined definition of antibiotic appropriateness, and detailed data at both the antibiotic and patient level. However, the study was limited by variable patient enrollment and hospital participation, meaning that the results could be more representative of larger hospitals or those that participated in more surveys. Another limitation is that ID consultation was defined as having an ID consultant note in the chart in the past 7 days. Thus, some antibiotic orders may have been entered after ID consultation was complete, resulting in an incorrect association of ID consultation with the appropriateness outcome for such orders. There may also be bias by ASP providers to judge orders reviewed by ID more favorably. Lastly, the determination of antibiotic inappropriateness used only data from chart review. Even with a provided definition of “inappropriate,” ASP teams may have judged orders differently due to varying expertise and institutional practices, and chart review is not always sufficient to adequately understand clinician rationale.

In summary, inappropriate antibiotic prescribing remains common among hospitalized children and varies by clinical service. By exploring inappropriateness across antibiotic type and clinical service, we enabled identification of more focused areas for ASP intervention. This study specifically highlighted opportunities for improvement among surgical specialties with prolonged or unnecessary surgical prophylaxis and for the PICU and hospitalists with the use of broad-spectrum antibiotics and overtreatment of infections. ASPs should consider stratifying antibiotic use data by clinical services to further refine and target ASP guidelines and interventions.

## Supplementary material

For supplementary material accompanying this paper visit https://doi.org/10.1017/ice.2023.56. click here to view supplementary material
