# Supplementary material for: Appropriateness of antibiotic prescribing varies by clinical services at United States children’s hospitals
Source: Infect Control Hosp Epidemiol. 2023 Apr 12;44(11):1711–7. doi: 10.1017/ice.2023.56 (PMC10665883; doi:10.1017/ice.2023.56)
Supplement: Supplementary file 1 [file S0899823X23000569sup001.docx]

**Supplementary Table 1. Reasons for Inappropriate Antibiotic Use**

| 1. Treatment for infection is not indicated |
| --- |
| 2. Treatment for infection was indicated, but duration is too long |
| 3. Prophylaxis for infection is not indicated |
| 4. Unnecessary prolonged surgical prophylaxis (e.g. clean and clean contaminated cases beyond single dose, tube prophylaxis, etc.) |
| 5. Unnecessary duplicate therapy (e.g. double anti-anaerobic coverage, double gram-negative coverage for infections that are not CREs) |
| 6. Spectrum is too broad empirically (no culture data available) |
| 7. Spectrum is too broad based on culture/susceptibility or rapid diagnostics |
| 8. Spectrum is too narrow empirically (drug does not cover all likely pathogens that require treatment) |
| 9. Spectrum is too narrow based on culture/susceptibility or rapid diagnostics (drug is not covering identified organisms that require treatment) |
| 10. Drug is highly bioavailable (fluoroquinolones, linezolid, clindamycin, doxycycline, TMP-SMX, rifampin, azithromycin) and administered IV, but child could take enterally because s/he is taking enteral feeds and/or another enteral medication |
| 11. Route is incorrect for indication |
| 12. Not preferred agent for indication (e.g. poor CNS penetration) |
| 13. Indication for use is unclear and use is likely unnecessary based on chart review (if you would not feel comfortable telling providers to discontinue the drug without additional information, do not consider this a reason for inappropriate use) |
| 14. Other |

*Note.* Abbreviations: CREs, carbapenem-resistant Enterobacterales; TMP-SMX, trimethoprim-sulfamethoxazole; IV, intravenously; CNS, central nervous system.

**Supplementary Table 2. Patient Demographic Data Stratified by Year**

| Variable | 2019 Patients,  N = 6,588^a^ | 2020 Patients,  N = 2,675^a^ | All Patients,  N = 9,263^a^ |
| --- | --- | --- | --- |
| *Patient age, y, median (IQR)* | 4.0 (0.0, 12.0) | 4.0 (0.0, 12.0) | 4.0 (0.0, 12.0) |
| *Sex* |  |  |  |
| Female | 3,027 (46%) | 1,257 (47%) | 4,284 (46%) |
| Male | 3,526 (54%) | 1,411 (53%) | 4,937 (54%) |
| *Clinical service* |  |  |  |
| CICU | 427 (6.5%) | 200 (7.5%) | 627 (6.8%) |
| Hospitalists | 1,256 (19%) | 469 (18%) | 1,725 (19%) |
| General Surgery | 444 (6.8%) | 192 (7.2%) | 636 (6.9%) |
| Hematology/Oncology | 1,176 (18%) | 576 (22%) | 1,752 (19%) |
| Medical Subspecialties | 1,008 (15%) | 332 (12%) | 1,340 (15%) |
| NICU | 818 (13%) | 348 (13%) | 1,166 (13%) |
| PICU | 964 (15%) | 383 (14%) | 1,347 (15%) |
| Surgical Subspecialties | 431 (6.6%) | 163 (6.1%) | 594 (6.5%) |
| *Ventilation status* |  |  |  |
| Invasive Ventilation | 1,111 (17%) | 501 (19%) | 1,612 (17%) |
| No Ventilation | 4,537 (69%) | 1,834 (69%) | 6,371 (69%) |
| Non-Invasive Ventilation | 897 (14%) | 325 (12%) | 1,222 (13%) |
| Unknown | 17 (0.3%) | 5 (0.2%) | 22 (0.2%) |
| *Infectious disease consult* | 1,290 (20%) | 601 (23%) | 1,891 (21%) |
| *Immunocompromised status* | 1,696 (26%) | 787 (29%) | 2,483 (27%) |
| Abbreviations: IQR, interquartile range; CICU, cardiac intensive care unit; NICU, neonatal intensive care unit; PICU, pediatric intensive care unit.  ^a^Median (IQR); n (%) | | | |

**Supplementary Table 3. Antibiotic Orders Stratified by Year**

| Variable | 2019 Orders  N = 9,532^a^ | 2020 Orders  N = 3,812^a^ | All Orders  N = 13,344^a^ |
| --- | --- | --- | --- |
| *Antibiotic Classification* |  |  |  |
| 1G Cephalosporin | 679 (7.1%) | 301 (7.9%) | 980 (7.3%) |
| 2G Cephalosporin | 38 (0.4%) | 28 (0.7%) | 66 (0.5%) |
| 3G Cephalosporin - IV | 983 (10%) | 355 (9.3%) | 1,338 (10%) |
| 3G Cephalosporin - PO | 44 (0.5%) | 18 (0.5%) | 62 (0.5%) |
| Aminoglycoside | 631 (6.6%) | 195 (5.1%) | 826 (6.2%) |
| Aminopenicillin-BLI | 370 (3.9%) | 165 (4.3%) | 535 (4.0%) |
| Anti-pseudomonal beta-lactam | 1,615 (17%) | 634 (17%) | 2,249 (17%) |
| Broad-spectrum GP agent | 103 (1.1%) | 32 (0.8%) | 135 (1.0%) |
| Carbapenem | 265 (2.8%) | 121 (3.2%) | 386 (2.9%) |
| Clindamycin | 416 (4.4%) | 181 (4.7%) | 597 (4.5%) |
| Fluoroquinolone | 312 (3.3%) | 164 (4.3%) | 476 (3.6%) |
| Macrolide | 264 (2.8%) | 51 (1.3%) | 315 (2.4%) |
| Metronidazole | 496 (5.2%) | 221 (5.8%) | 717 (5.4%) |
| Miscellaneous | 188 (2.0%) | 60 (1.6%) | 248 (1.9%) |
| Narrow-spectrum penicillin | 1,201 (13%) | 411 (11%) | 1,612 (12%) |
| Sulfamethoxazole and trimethoprim | 1,099 (12%) | 526 (14%) | 1,625 (12%) |
| Tetracycline | 75 (0.8%) | 26 (0.7%) | 101 (0.8%) |
| Vancomycin | 753 (7.9%) | 323 (8.5%) | 1,076 (8.1%) |
| *Note.* Abbreviations: 3G, third-generation; IV, intravenous; 1G, first-generation; BLI, beta-lactam inhibitor; GP, gram-positive; 2G, second-generation; PO, oral.  ^a^ n (%) | | | |

**Supplementary Table 4. Antibiotic Classifications**

| Antibiotic Classification | Individual Antibiotic |
| --- | --- |
| *1G Cephalosporin* |  |
|  | Cefazolin |
|  | Cephalexin |
| *2G Cephalosporin* |  |
|  | Cefotetan |
|  | Cefoxitin |
|  | Cefprozil |
|  | Cefuroxime |
| *3G Cephalosporin - IV* |  |
|  | Cefotaxime |
|  | Ceftriaxone |
| *3G Cephalosporin - PO* |  |
|  | Cefdinir |
|  | Cefixime |
| *Aminoglycoside* |  |
|  | Amikacin |
|  | Gentamicin |
|  | Neomycin |
|  | Tobramycin |
| *Aminopenicillin-BLI* |  |
|  | Amoxicillin and enzyme inhibitor |
|  | Ampicillin and enzyme inhibitor |
| *Anti-pseudomonal beta-lactam* |  |
|  | Aztreonam |
|  | Cefepime |
|  | Ceftazidime |
|  | Piperacillin |
|  | Piperacillin and enzyme inhibitor |
| *Broad-spectrum GP agent* |  |
|  | Ceftaroline fosamil |
|  | Daptomycin |
|  | Linezolid |
|  | Tidezolid |
| *Carbapenem* |  |
|  | Ertapenem |
|  | Imipenem/cilastatin |
|  | Meropenem |
| *Clindamycin* |  |
|  | Clindamycin |
| *Fluoroquinolone* |  |
|  | Ciprofloxacin |
|  | Delafloxacin |
|  | Levofloxacin |
|  | Moxifloxacin |
| *Macrolide* |  |
|  | Azithromycin |
|  | Clarithromycin |
|  | Erythromycin |
| *Metronidazole* |  |
|  | Metronidazole |
| *Miscellaneous* |  |
|  | Ceftazidime/avibactam |
|  | Ceftolozane/tazobactam |
|  | Colistin |
|  | Ethambutol |
|  | Ethionamide |
|  | Fosfomycin |
|  | Isoniazid |
|  | Nitrofurantoin |
|  | Pyrazinamide |
|  | Rifabutin |
|  | Rifampicin |
|  | Rifampin |
|  | Rifaximin |
|  | Sulfamethoxazole |
|  | Sulfonamides & Trimethoprim |
|  | Tigecycline |
| *Narrow-spectrum penicillin* |  |
|  | Amoxicillin |
|  | Ampicillin |
|  | Benzathine benzylpenicillin |
|  | Benzylpenicillin |
|  | Nafcillin |
|  | Oxacillin |
|  | Penicillin VK |
|  | Procaine benzylpenicillin |
| *Sulfamethoxazole and trimethoprim* |  |
|  | Sulfamethoxazole and trimethoprim |
| *Tetracycline* |  |
|  | Doxycycline |
|  | Minocycline |
|  | Tetracycline |
| *Vancomycin* |  |
|  | Vancomycin |

*Note.* Abbreviations: 3G, third-generation; IV, intravenous; 1G, first-generation; BLI, beta-lactam inhibitor; GP, gram-positive; 2G, second-generation; PO, oral.

**Supplementary Table 5. Clinical Service Groups**

| Service Group | Individual Clinical Service |
| --- | --- |
| *CICU* |  |
|  | CICU |
| *General Surgery* |  |
|  | General Surgery |
| *Hematology/Oncology* |  |
|  | Bone Marrow Transplant |
|  | Hematology |
|  | Neuro-oncology |
|  | Oncology |
| *Hospitalists* |  |
|  | General Pediatrics |
|  | Well-baby Nursery |
| *Medical Subspecialties* |  |
|  | Adolescent Medicine |
|  | Allergy/Immunology |
|  | Cardiology / (non-CICU) |
|  | Endocrinology |
|  | Gastroenterology |
|  | Infectious Disease |
|  | Nephrology |
|  | Neurology |
|  | Psychiatry |
|  | Pulmonary |
|  | Rehabilitation |
|  | Rheumatology |
|  | Solid Organ Transplant |
| *NICU* |  |
|  | NICU |
| *PICU* |  |
|  | PICU |
| *Surgical Subspecialties* |  |
|  | Cardiovascular Surgery (non-CICU) |
|  | Neurosurgery |
|  | Orthopedics |
|  | Otolaryngology / ENT |
|  | Plastic Surgery |
|  | Urology |

*Note.* Abbreviations: CICU, cardiac intensive care unit; NICU, neonatal intensive care unit; PICU, pediatric intensive care unit.

**Supplementary Table 6. Inappropriate Antibiotic Orders Stratified by Clinical Service and Antibiotic Classification**

| Clinical Service | Antibiotic Classification | Percentage of Inappropriate Use  (inappropriate/total orders) |
| --- | --- | --- |
| CICU | 1G Cephalosporin | 19.1% (30/157) |
| CICU | 3G Cephalosporin - IV | 22.2% (10/45) |
| CICU | Aminoglycoside | 6.5% (2/31) |
| CICU | Aminopenicillin-BLI | 7.1% (1/14) |
| CICU | Anti-pseudomonal beta-lactam | 16.5% (33/200) |
| CICU | Broad-spectrum GP agent | 21.4% (3/14) |
| CICU | Carbapenem | 41.9% (13/31) |
| CICU | Clindamycin | 25.0% (4/16) |
| CICU | Fluoroquinolone | 21.4% (3/14) |
| CICU | Macrolide | 33.3% (2/6) |
| CICU | Metronidazole | 15.0% (3/20) |
| CICU | Miscellaneous | 5.9% (1/17) |
| CICU | Narrow-spectrum penicillin | 6.0% (7/117) |
| CICU | Sulfamethoxazole and trimethoprim | 10.6% (9/85) |
| CICU | Tetracycline | 50.0% (1/2) |
| CICU | Vancomycin | 15.7% (18/115) |
| General Surgery | 1G Cephalosporin | 29.5% (18/61) |
| General Surgery | 2G Cephalosporin | 21.4% (6/28) |
| General Surgery | 3G Cephalosporin - IV | 4.3% (10/232) |
| General Surgery | Aminoglycoside | 38.5% (5/13) |
| General Surgery | Aminopenicillin-BLI | 30.3% (10/33) |
| General Surgery | Anti-pseudomonal beta-lactam | 22.4% (38/170) |
| General Surgery | Broad-spectrum GP agent | 14.3% (1/7) |
| General Surgery | Carbapenem | 21.4% (3/14) |
| General Surgery | Clindamycin | 38.1% (8/21) |
| General Surgery | Fluoroquinolone | 30.4% (7/23) |
| General Surgery | Metronidazole | 9.0% (25/279) |
| General Surgery | Miscellaneous | 9.1% (1/11) |
| General Surgery | Narrow-spectrum penicillin | 25.9% (7/27) |
| General Surgery | Sulfamethoxazole and trimethoprim | 26.3% (5/19) |
| General Surgery | Vancomycin | 23.5% (4/17) |
| Hematology/Oncology | 1G Cephalosporin | 39.3% (11/28) |
| Hematology/Oncology | 2G Cephalosporin | 100.0% (1/1) |
| Hematology/Oncology | 3G Cephalosporin - IV | 15.3% (22/144) |
| Hematology/Oncology | 3G Cephalosporin - PO | 21.4% (3/14) |
| Hematology/Oncology | Aminoglycoside | 38.9% (7/18) |
| Hematology/Oncology | Aminopenicillin-BLI | 11.1% (3/27) |
| Hematology/Oncology | Anti-pseudomonal beta-lactam | 6.2% (42/678) |
| Hematology/Oncology | Broad-spectrum GP agent | 12.5% (2/16) |
| Hematology/Oncology | Carbapenem | 19.6% (22/112) |
| Hematology/Oncology | Clindamycin | 30.2% (16/53) |
| Hematology/Oncology | Fluoroquinolone | 7.2% (17/235) |
| Hematology/Oncology | Macrolide | 8.8% (6/68) |
| Hematology/Oncology | Metronidazole | 21.4% (15/70) |
| Hematology/Oncology | Miscellaneous | 14.3% (3/21) |
| Hematology/Oncology | Narrow-spectrum penicillin | 8.6% (12/140) |
| Hematology/Oncology | Sulfamethoxazole and trimethoprim | 0.9% (8/884) |
| Hematology/Oncology | Vancomycin | 11.9% (26/219) |
| Hospitalists | 1G Cephalosporin | 12.2% (19/156) |
| Hospitalists | 3G Cephalosporin - IV | 14.9% (61/409) |
| Hospitalists | 3G Cephalosporin - PO | 43.5% (10/23) |
| Hospitalists | Aminoglycoside | 12.3% (9/73) |
| Hospitalists | Aminopenicillin-BLI | 13.1% (22/168) |
| Hospitalists | Anti-pseudomonal beta-lactam | 19.0% (29/153) |
| Hospitalists | Broad-spectrum GP agent | 5.3% (1/19) |
| Hospitalists | Carbapenem | 9.3% (4/43) |
| Hospitalists | Clindamycin | 19.6% (53/271) |
| Hospitalists | Fluoroquinolone | 17.0% (8/47) |
| Hospitalists | Macrolide | 19.0% (16/84) |
| Hospitalists | Metronidazole | 20.4% (19/93) |
| Hospitalists | Miscellaneous | 5.7% (3/53) |
| Hospitalists | Narrow-spectrum penicillin | 9.3% (33/356) |
| Hospitalists | Sulfamethoxazole and trimethoprim | 10.6% (10/94) |
| Hospitalists | Tetracycline | 3.2% (1/31) |
| Hospitalists | Vancomycin | 12.5% (16/128) |
| Medical Subspecialties | 1G Cephalosporin | 16% (13/81) |
| Medical Subspecialties | 3G Cephalosporin - IV | 23.2% (29/125) |
| Medical Subspecialties | 3G Cephalosporin - PO | 43.8% (7/16) |
| Medical Subspecialties | Aminoglycoside | 10.9% (28/257) |
| Medical Subspecialties | Aminopenicillin-BLI | 16.3% (14/86) |
| Medical Subspecialties | Anti-pseudomonal beta-lactam | 12.7% (43/339) |
| Medical Subspecialties | Broad-spectrum GP agent | 20.5% (9/44) |
| Medical Subspecialties | Carbapenem | 15.9% (10/63) |
| Medical Subspecialties | Clindamycin | 25.7% (18/70) |
| Medical Subspecialties | Fluoroquinolone | 24.0% (18/75) |
| Medical Subspecialties | Macrolide | 13.7% (10/73) |
| Medical Subspecialties | Metronidazole | 25.4% (16/63) |
| Medical Subspecialties | Miscellaneous | 18.4% (14/76) |
| Medical Subspecialties | Narrow-spectrum penicillin | 5.8% (12/206) |
| Medical Subspecialties | Sulfamethoxazole and trimethoprim | 6.4% (19/295) |
| Medical Subspecialties | Tetracycline | 10.3% (4/39) |
| Medical Subspecialties | Vancomycin | 15.3% (26/170) |
| NICU | 1G Cephalosporin | 22.5% (25/111) |
| NICU | 2G Cephalosporin | 25.0% (3/12) |
| NICU | 3G Cephalosporin - IV | 11.1% (4/36) |
| NICU | Aminoglycoside | 6.3% (22/351) |
| NICU | Aminopenicillin-BLI | 15.8% (3/19) |
| NICU | Anti-pseudomonal beta-lactam | 14.5% (44/303) |
| NICU | Broad-spectrum GP agent | 25.0% (1/4) |
| NICU | Carbapenem | 16% (4/25) |
| NICU | Clindamycin | 11.4% (4/35) |
| NICU | Metronidazole | 3.9% (3/76) |
| NICU | Narrow-spectrum penicillin | 5.8% (34/586) |
| NICU | Sulfamethoxazole and trimethoprim | 10.4% (5/48) |
| NICU | Vancomycin | 17.7% (25/141) |
| PICU | 1G Cephalosporin | 27.5% (33/120) |
| PICU | 2G Cephalosporin | 16.7% (2/12) |
| PICU | 3G Cephalosporin - IV | 24.7% (73/296) |
| PICU | 3G Cephalosporin - PO | 60.0% (3/5) |
| PICU | Aminoglycoside | 25.9% (21/81) |
| PICU | Aminopenicillin-BLI | 21.7% (28/129) |
| PICU | Anti-pseudomonal beta-lactam | 17.7% (61/344) |
| PICU | Broad-spectrum GP agent | 28.6% (6/21) |
| PICU | Carbapenem | 23.1% (21/91) |
| PICU | Clindamycin | 30.3% (27/89) |
| PICU | Fluoroquinolone | 22.2% (14/63) |
| PICU | Macrolide | 23.8% (15/63) |
| PICU | Metronidazole | 11.2% (10/89) |
| PICU | Miscellaneous | 18.0% (9/50) |
| PICU | Narrow-spectrum penicillin | 11.5% (16/139) |
| PICU | Sulfamethoxazole and trimethoprim | 5.9% (10/169) |
| PICU | Tetracycline | 13.3% (2/15) |
| PICU | Vancomycin | 14.7% (32/217) |
| Surgical Subspecialties | 1G Cephalosporin | 28.9% (80/277) |
| Surgical Subspecialties | 3G Cephalosporin - IV | 19.1% (9/47) |
| Surgical Subspecialties | 3G Cephalosporin - PO | 75% (3/4) |
| Surgical Subspecialties | Aminoglycoside | 37.5% (3/8) |
| Surgical Subspecialties | Aminopenicillin-BLI | 24.1% (13/54) |
| Surgical Subspecialties | Anti-pseudomonal beta-lactam | 13.3% (10/75) |
| Surgical Subspecialties | Carbapenem | 12.5% (1/8) |
| Surgical Subspecialties | Clindamycin | 20.5% (9/44) |
| Surgical Subspecialties | Fluoroquinolone | 7.7% (1/13) |
| Surgical Subspecialties | Macrolide | 20.0% (1/5) |
| Surgical Subspecialties | Metronidazole | 42.3% (11/26) |
| Surgical Subspecialties | Miscellaneous | 8.3% (1/12) |
| Surgical Subspecialties | Narrow-spectrum penicillin | 20.5% (9/44) |
| Surgical Subspecialties | Sulfamethoxazole and trimethoprim | 4.0% (1/25) |
| Surgical Subspecialties | Vancomycin | 14.7% (10/68) |

*Note.* Abbreviations: 1G, first-generation; 2G, second-generation; 3G, third-generation; BLI, beta-lactam inhibitor; CICU, cardiac intensive care unit; GP, gram-positive; IV, intravenous; PICU, pediatric intensive care unit; PO, oral.
